# A comprehensive diagnostic model for tuberculous PE: integration of clinical and immunological biomarkers

**DOI:** 10.3389/fpubh.2026.1777931

**Published:** 2026-03-27

**Authors:** Tingting Li, Huanqing Liu, Guolian Zhao, Qian Lei, Zhuhong You, Jianying Li

**Affiliations:** 1 Drug Clinical Trial Institution Office, Xi'an Chest Hospital, Xi'an, Shaanxi, China; 2 Information Management Office, Northwestern Polytechnical University, Xi'an, Shaanxi, China; 3 Laboratory Department, Xi'an Chest Hospital, Xi'an, Shaanxi, China; 4 Department of Pharmacy, Xi'an Chest Hospital, Xi'an, Shaanxi, China; 5 School of Computer Science, Northwestern Polytechnical University, Xi'an, Shaanxi, China

**Keywords:** adenosine deaminase, diagnostic markers, predictive model, T-SPOT.TB, tuberculous PE

## Abstract

**Background:**

Tuberculous pleurisy represents a prevalent form of extrapulmonary tuberculosis and constitutes a significant diagnostic challenge in clinical practice, particularly in endemic regions where it accounts for approximately 20–30% of all PEs. The nonspecific clinical presentation and the limitations of conventional diagnostic methods—including the low sensitivity and prolonged turnaround time of pleural fluid culture—often result in delayed diagnosis and treatment, potentially compromising patient outcomes.

**Objective:**

To develop and validate a comprehensive, evidence-based predictive model that integrates readily available clinical and immunological biomarkers to enhance the early and accurate diagnosis of tuberculous pleurisy, thereby supporting timely clinical decision-making and optimized patient management.

**Methods:**

We conducted a retrospective cohort study of 523 consecutive patients presenting with PE at a tertiary care center between 2010 and 2021, including 375 patients with confirmed tuberculous pleurisy and 148 with non-tuberculous effusions. Demographics, biochemical markers (adenosine deaminase, lactate dehydrogenase, C-reactive protein, D-dimer), immunological parameters (T-SPOT.TB, T-cell subsets), and clinical indicators were systematically evaluated. Statistical analyses encompassed descriptive and comparative tests, multivariate logistic regression, and receiver operating characteristic curve analysis. Missing data were addressed using median imputation (Rivalta test, 4.4%) and case-wise deletion (T-cell subsets, 14.9%).

**Results:**

Five independent predictors were significantly associated with tuberculous pleurisy: younger age, elevated adenosine deaminase, lower C-reactive protein, positive Rivalta test, and positive T-SPOT.TB result. The multivariate logistic regression model demonstrated excellent discriminative performance (pseudo *R*^2^ = 0.450) and strong model fit. Receiver operating characteristic analysis identified an optimal adenosine deaminase cutoff of 30.5 U/L, yielding a sensitivity of 7.1%. Model robustness was further confirmed through rigorous internal validation.

**Conclusion:**

This study presents a robust and clinically applicable diagnostic model that effectively distinguishes tuberculous pleurisy from non-tuberculous PEs by integrating multiple routinely available biomarkers. The model offers a practical, cost-effective tool for early diagnosis, with the potential to improve therapeutic timeliness and patient outcomes across varied healthcare settings.

## Introduction

Tuberculous pleurisy (TBP) is caused by Mycobacterium tuberculosis infection of the pleura and is characterized by chronic effusion and a large accumulation of inflammatory cells in the pleural cavity (1). TBP is the main cause of PE (PE) and one of the most common types of extrapulmonary tuberculosis, constituting a prominent public health problem in developing countries including China (2, 3). The diagnostic complexity of TBP arises from its remarkably heterogeneous clinical presentation, which often mimics other pleural pathologies, and the inherent limitations of traditional diagnostic approache (4). This diagnostic uncertainty frequently leads to delayed therapeutic intervention, potentially resulting in increased morbidity, prolonged hospitalization, and suboptimal patient outcomes. The gold standard for diagnosing TPE is to detect Mycobacterium tuberculosis in PE or pleural biopsy specimens (2). However, the positive rate of pleural fluid microbial culture is low and time-consuming, usually taking up to 8 weeks (5). Furthermore, obtaining pleural specimens through thoracoscopy or percutaneous pleural biopsy requires surgical operation, which poses a risk of severe trauma and complications, such as iatrogenic pneumothorax (6). Emerging technologies like metagenomic next-generation sequencing (mNGS) showed promise, with recent studies reporting >80% sensitivity for pathogen identification in PE, including drug-resistant TB strains (7), however, these methods remain costly and inaccessible in resource-limited settings, where TBP burden is highest. Therefore, diagnosing TBP remains challenging. This highlights the urgent need for an early TBP diagnosis method that is less invasive, more accurate and more cost-effective. According to WHO Global TB Reports on reported TB in the USA, pleural TB is among the most common forms of extrapulmonary tuberculosis, accounting for approximately 20–30% of extrapulmonary TB cases (8).

Recent advances have highlighted the role of immunological and biochemical markers in improving TBP diagnosis. Adenosine deaminase (ADA) are the most extensively studied, with pooled sensitivities of 88–92% and 89–94% specificity in meta-analyses (9). However, studies have found that the cut-off values for diagnosing tuberculous pleurisy using ADA detection for thoracic effusion vary greatly and there is no definite conclusion yet (10, 11). ADA testing is also affected by factors such as immune status, age (12). The efficacy evaluation of anti-tuberculosis treatment mostly relies on clinical symptoms and imaging manifestations, lacking objective and dynamic test indicators (13). For example, the absorption rate of PE is affected by the degree of pleural adhesion (14), while the relationship between the dynamic changes of inflammatory markers such as C-reactive protein (CRP) and erythrocyte sedimentation rate (ESR) and the treatment response has not been systematically verified (15). The prevalence of drug-resistant Mycobacterium tuberculosis (with a rifampicin resistance rate of approximately 8.7%) further increases the risk of treatment failure (16), and there is an urgent need for an early warning model based on test data. Recent studies suggest that the combination of ADA, lactate dehydrogenase (LDH) can improve diagnostic efficacy (17), but most of the existing models are based on small sample studies (18), and lack longitudinal analysis of the dynamic changes of indicators during the treatment process (19). Complementing traditional biochemical markers, interferon-gamma release assays (IGRAs), particularly T-SPOT.TB, have shown considerable promise in improving diagnostic accuracy (20). These assays detect T-cell responses to Mycobacterium tuberculosis-specific antigens, offering superior specificity compared to tuberculin skin testing while maintaining high sensitivity (20). The integration of IGRAs with traditional biomarkers has the potential to create a more robust diagnostic framework for TBP. Despite these technological advances, the optimal combination of diagnostic markers and their relative importance in clinical practice remains incompletely elucidated. Furthermore, the absence of comprehensive microbiological diagnosis represents a critical missed opportunity for patients, as it increases the risk of underdiagnosing drug-resistant tuberculosis, which is increasingly prevalent in endemic regions (21). Additionally, pleural intervention is frequently required for therapeutic purposes, including the evacuation of symptomatic effusions and the prevention of pleural complications (22). This study aims to address these critical gaps by systematically evaluating clinical and immunological predictors of TBP in a large, real-world cohort, providing evidence-based guidance for clinical decision-making while appropriately addressing the challenges of missing data. Our comprehensive approach integrates multiple diagnostic modalities to develop a robust predictive model that serves as a complementary diagnostic tool, enhancing rather than replacing microbiological diagnosis for comprehensive patient care.

## Methods

### Object of study

A total of 523 patients with unexplained PE admitted to Xi 'an Chest Hospital from January 2020 to December 2021 were retrospectively collected as the research objects. Inclusion criteria: Age ≥18 years old; Complete clinical data; All subjects underwent peripheral blood T-SPOT.TB, chest CT scan or chest ultrasound examination within 3 days after admission, and PE was collected for routine and biochemical detection. The diagnosis was clear. Exclusion criteria: Complicated with other serious diseases; Incomplete data; Insufficient follow-up time. Patients with missing test results, HIV positive, immunosuppressed, and non-tuberculous mycobacterial infection (23).

(By “clear diagnosis,” a definitive final diagnosis (for both TBP and non-TBP groups) was established based on a composite reference standard integrating clinical presentation, laboratory results (including microbiology and pathology where available), imaging findings, and response to treatment or clinical follow-up.)

### Composition of the non-tuberculous PE (non-TBP) control group

To ensure the clinical relevance of our diagnostic model, the non-TBP control group (*n* = 148) was explicitly composed to represent the spectrum of diseases that constitute the primary differential diagnoses for TBP in clinical practice. This group included patients with malignant PE, parapneumonic effusion/empyema, effusions secondary to autoimmune diseases (e.g., rheumatoid arthritis, systemic lupus erythematosus), and a smaller proportion of transudative effusions (e.g., due to heart failure, cirrhosis, or nephrotic syndrome). This composition ensures that the model's performance is evaluated against the challenge of distinguishing TBP from other clinically significant exudative effusions, rather than from straightforward transudates.

Diagnostic criteria: (1) patients with fever, cough, fatigue, night sweats and other symptoms, imaging showed PE, or PE was significantly absorbed after anti-tuberculosis drug treatment; (2) Positive culture of Mycobacterium tuberculosis in PE; (3) The molecular biology test of Mycobacterium tuberculosis in PE was positive; (4) Pleural biopsy was positive for Mycobacterium tuberculosis. Patients who met one of (1) and (2)–(4) were diagnosed as tuberculous pleurisy. Other causes of PE are non-tuberculous pleurisy, including infectious PE and malignant PE.

Pulmonary tuberculosis (PTB) screening: All enrolled patients underwent screening for concomitant pulmonary tuberculosis through chest CT or chest ultrasound within 3 days of admission. Peripheral blood T-SPOT.TB testing was performed as a laboratory indicator of TB infection. Clinical assessment for respiratory symptoms (cough, fever, night sweats) was part of the diagnostic workup.

### Clinical data collection

The following clinical indicators of the patients were collected: ADA (U/L), LDH (U/L), CRP (mg/L), Rivalta test results, WBC count (10^6^/L), TSPOT.TB results of peripheral blood samples, CD3 level (%), CD4 level (%), CD4/CD8 ratio, D-dimer level (μg/L) levels.

### Statistical analysis

Descriptive statistics were calculated for all variables. Continuous variables were compared using independent *t*-tests; categorical variables using chi-square tests. Variables with *P* < 0.1 in univariate analysis were entered into multivariable logistic regression to identify independent predictors. Model discrimination was evaluated using the area under the receiver operating characteristic curve; calibration was assessed via the Hosmer-Lemeshow test. Statistical significance was set at *P* < 0.05. All analyses were performed using Python 3.8 with pandas, scipy, and statsmodels. Missing data handling: median imputation for Rivalta test; case-wise deletion for T-cell subset missing values. The detailed missing data analysis and sensitivity analysis results are reported in the Results section.

Internal Validation Procedure: To assess the model's generalizability and mitigate overfitting, we performed internal validation using 10-fold cross-validation. Specifically, the dataset (*n* = 445 for the final model) was randomly partitioned into 10 equally sized folds. The logistic regression model (with the five predefined predictors) was trained on 9 folds and validated on the remaining fold. This process was iterated 10 times, with each fold serving as the validation set exactly once. The performance metrics—including the area under the receiver operating characteristic curve (AUC), sensitivity, and specificity—were calculated for each validation fold and subsequently averaged to provide a final, robust estimate of the model's predictive performance.

Variables with *P* < 0.1 in univariate analysis were entered into a multivariable logistic regression model to identify independent predictors of TBP, with results reported as odds ratios (ORs) and 95% confidence intervals (CIs). Model discrimination was evaluated using the area under the receiver operating characteristic curve (AUC), and calibration was assessed via the Hosmer-Lemeshow test. Model performance was assessed using pseudo R-squared and likelihood ratio tests. Statistical significance was set at *P* < 0.05. All analyses were performed using Python 3.8 with pandas, scipy, and statsmodels packages.

## Results

### Baseline characteristics of the study population

A total of 523 patients with PE were included, comprising 375 TBP cases (71.7%) and 148 non-TBP cases (28.3%). The two groups were comparable in gender distribution (TBP: 67.5% male; non-TBP: 70.3% male; *P* = 0.495) and baseline comorbidities. However, significant differences were observed between TBP and non-TBP groups in several key variables. TBP patients were significantly younger than non-TBP patients (47.3 ± 18.9 vs 63.7 ± 20.1 years, *P* < 0.001).

Descriptive statistics were calculated for all variables. Continuous variables were compared using independent *t*-tests, while categorical variables were compared using chi-square tests. Missing data analysis revealed that 23 patients (4.4%) had missing Rivalta test results, while 78 patients (14.9%) had missing T-cell subset data. Missing data patterns were examined for potential bias. Rivalta test missingness was not significantly associated with group status (*P* = 0.156), suggesting that missing data were likely missing at random. T-cell subset missingness was also not significantly associated with group status (*P* = 0.234). Given the relatively low missing data rates and lack of significant association with outcome, the chosen imputation and deletion strategies were considered appropriate.

**Figure 1:**
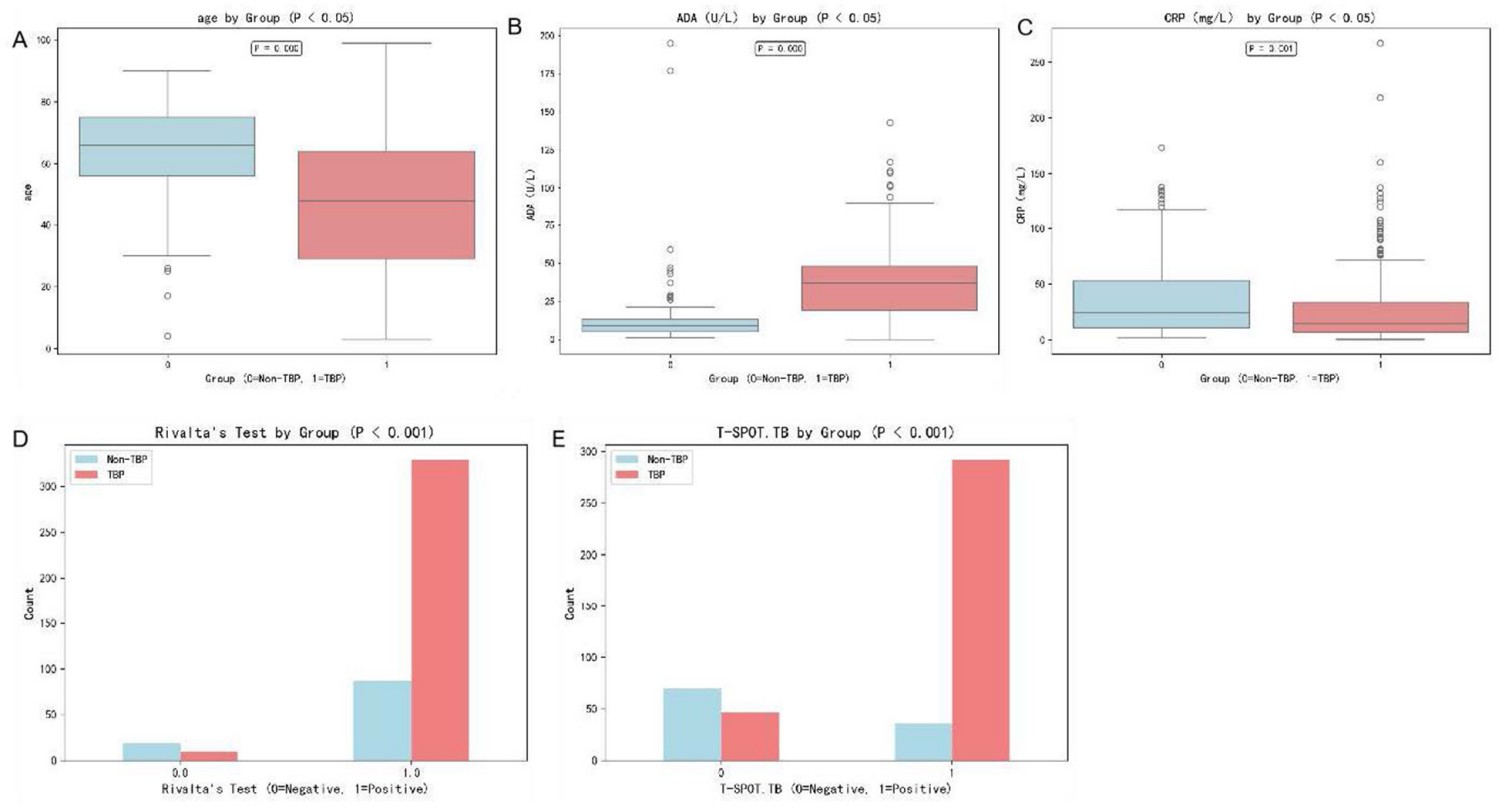
Boxplots of variables with significant differences between groups [**(A)**: Age distribution; **(B)**: ADA levels; **(C)**: CRP levels; **(D)**: Rivalta test results; **(E)**: T-SPOT.TB].

Key biomarkers exhibited marked differences (Table 1, Figure 1). ADA levels were markedly higher in TBP patients (37.3 ± 24.5 vs 14.2 ± 23.3 U/L, *P* < 0.001), while CRP levels were lower (28.2 ± 33.8 vs 40.3 ± 41.4 mg/L, *P* = 0.001). T-SPOT.TB positivity was significantly more common in TBP patients (86.1% vs 34.0%, *P* < 0.001), and Rivalta test positivity was also more frequent in TBP cases (97.1% vs 82.1%, *P* < 0.001). Other parameters including total protein, LDH, D-dimer, and WBC count also showed statistically significant differences between the two groups (all *P* < 0.01). Modest but significant variations were observed in T lymphocyte subsets (CD3^+^, CD4^+^, and CD4/CD8 ratio, all *P* < 0.05) (24).

**Table 1:** Baseline characteristics and group comparisons between TBP and non-TBP groups.

Variable	Overall (*n* = 523)	Non-TBP (*n* = 148)	TBP (*n* = 375)	*P*-value
Age (years)	51.6 ± 21.2	63.7 ± 20.1	47.3 ± 18.9	< 0.001
Gender (Male/Female)	357/166	104/44	253/122	0.495
Total protein (g/L)	563.0 ± 1,404.5	519.6 ± 1,522.8	580.1 ± 1,356.7	0.001
ADA (U/L)	30.8 ± 26.3	14.2 ± 23.3	37.3 ± 24.5	< 0.001
CRP (mg/L)	31.7 ± 36.5	40.3 ± 41.4	28.2 ± 33.8	0.001
LDH (U/L)	602.6 ± 1,402.4	535.4 ± 1,519.4	629.2 ± 1,354.6	0.002
D-dimer (μg/L)	3.8 ± 6.8	3.4 ± 5.6	4.0 ± 7.3	0.003
WBC sount (10^6^)	3,125.8 ± 6,447.5	2,496.0 ± 7,641.1	3,374.3 ± 5,903.6	0.004
T-SPOT.TB positive (%)	70.6	34.0	86.1	< 0.001
Rivalta's test positive (%)	88.5	82.1	97.1	< 0.001
CD3^+^ T cells (%)	888.6 ± 510.3	894.9 ± 498.3	886.7 ± 514.8	0.045
CD4^+^ T cells (%)	514.8 ± 452.9	569.8 ± 746.5	497.6 ± 308.4	0.023
CD4/CD8 ratio	1.61 ± 1.05	1.69 ± 1.01	1.59 ± 1.06	0.012

### Sensitivity analysis for missing data

To assess potential selection bias due to missing T-cell subset data, we conducted a sensitivity analysis comparing baseline characteristics between included (*n* = 445) and excluded (*n* = 78) cases (Table 2). No significant differences were observed in age (51.25 ± 21.30 vs 53.40 ± 20.93 years, *P* = 0.410), gender distribution (68.5% vs 66.7% male, *P* = 0.845), ADA levels (31.38 ± 25.42 vs 27.35 ± 30.89 U/L, *P* = 0.212), or CRP levels (30.57 ± 35.46 vs 37.86 ± 41.47 mg/L, *P* = 0.104) between the two groups. However, significant differences were observed in T-SPOT.TB positivity (73.7% vs 52.6%, *P* < 0.001) and TBP diagnosis rate (76.2% vs 46.2%, *P* < 0.001), which likely reflects the fact that T-cell subset testing was more frequently performed in patients with higher clinical suspicion of TBP. The lack of significant differences in key demographic and biochemical markers suggests that the exclusion of cases with missing T-cell data did not introduce substantial selection bias for the primary analysis.

**Table 2:** Comparison of baseline characteristics between patients included in the final multivariate model and those excluded due to missing T-cell subset data.

Characteristic	Patients included in final model (*n* = 445)	Patients excluded due to missing T-cell data (*n* = 78)	*P*-value
Demographics			
Age, years	51.25 ± 21.30	53.40 ± 20.93	0.410
Male gender, *n* (%)	305 (68.5%)	52 (66.7%)	0.845
Key diagnostic biomarkers			
ADA, U/L	31.38 ± 25.42	27.35 ± 30.89	0.212
CRP, mg/L	30.57 ± 35.46	37.86 ± 41.47	0.104
15.6-7.4,-14242ptT-SPOT.TB positive, *n* (%)	328 (73.7%)	41 (52.6%)	< 0.001
Outcome			
Final diagnosis of TBP, *n* (%)	339 (76.2%)	36 (46.2%)	< 0.001

ADA, adenosine deaminase; CRP, C-reactive protein; TBP, tuberculous pleurisy.

Data are presented as mean ± standard deviation for continuous variables and number (percentage) for categorical variables. *P*-values were derived from independent *t*-tests for continuous variables and chi-square tests for categorical variables. A *P*-value < 0.05 was considered statistically significant.

### Correlation analysis of diagnostic biomarkers

**Figure 2:**
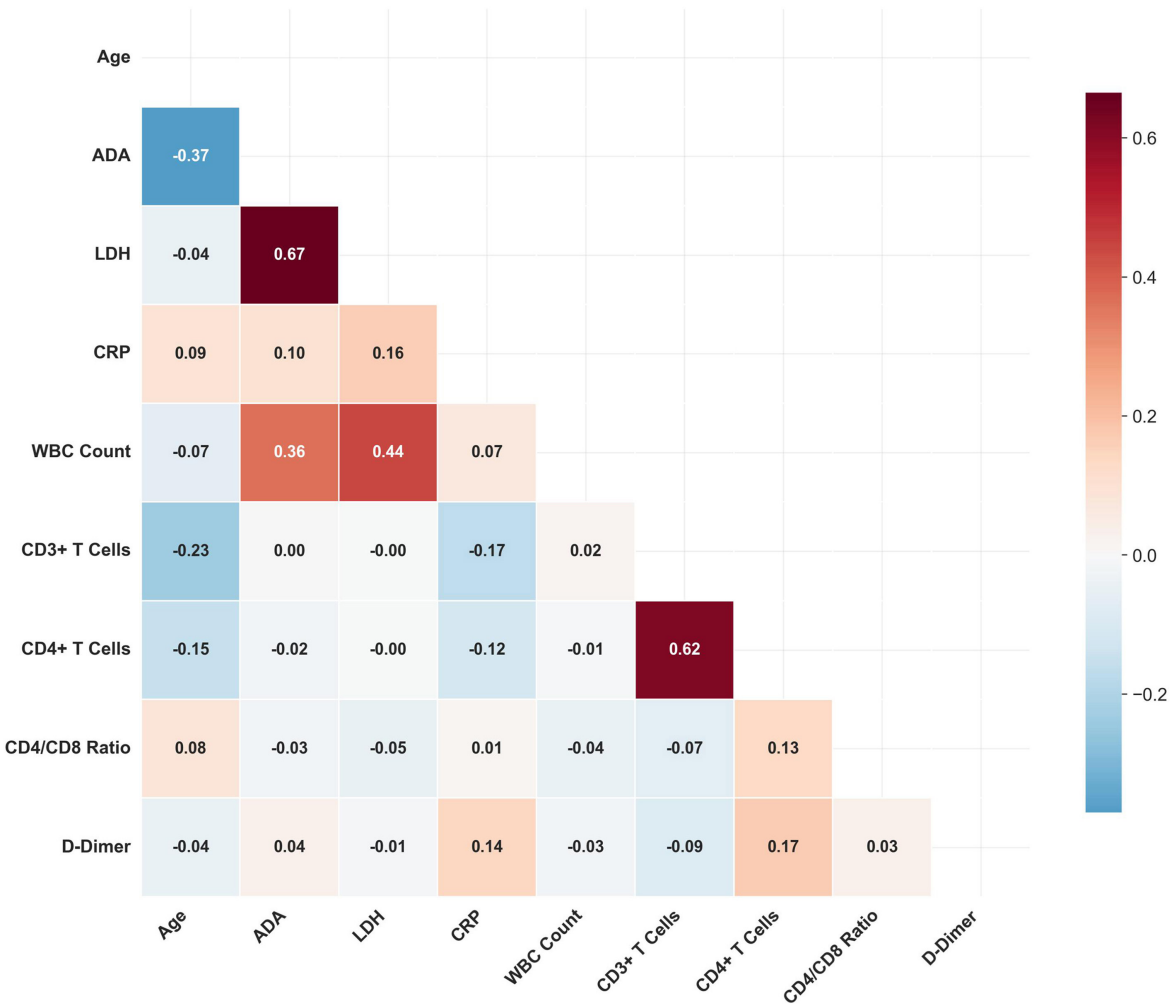
Correlation matrix of clinical and laboratory variables. (The color scale represents correlation strength, with red indicating positive correlations and blue indicating negative correlations).

Correlation matrix analysis was performed to elucidate the relationships among the studied diagnostic and immunological variables (Figure 2). The most pronounced finding was a strong positive correlation between LDH and ADA levels (*r* = 0.67, *P* < 0.001), indicating a close pathophysiological link between these enzymes in the context of PE. Immunologically, as anticipated, a strong positive correlation was identified between CD4^+^ T-cell counts and total CD3^+^ T-cell counts (*r* = 0.62, *P* < 0.001), validating the consistency of our flow cytometric immunophenotyping. Moderate positive correlations were observed between the WBC count and both LDH (*r* = 0.44, *P* < 0.001) and ADA (*r* = 0.36, *P* < 0.001), reinforcing the interplay between cellular inflammation and enzymatic biomarkers. A clinically significant moderate negative correlation was found between patient age and ADA levels (*r* = −0.37, *P* < 0.001), suggesting that diagnostic interpretations of ADA may need to account for patient age. Critically, T-cell subsets (CD3^+^ and CD4^+^) demonstrated negligible correlations with key biochemical markers such as ADA (*r* = 0.00 and *r* = −0.02, respectively) and LDH (*r* = −0.00). This statistical independence underscores the potential of T-cell immunology to provide complementary diagnostic information beyond traditional assays. Other notable associations included a weak positive correlation between D-dimer and CD4^+^ T-cell counts (*r* = 0.17, *P* = 0.02), hinting at a connection between coagulation and immune activation, and a weak correlation between the CD4/CD8 ratio and CD4+ counts (*r* = 0.13, *P* = 0.05).

### Receiver operating characteristic analysis

**Figure 3:**
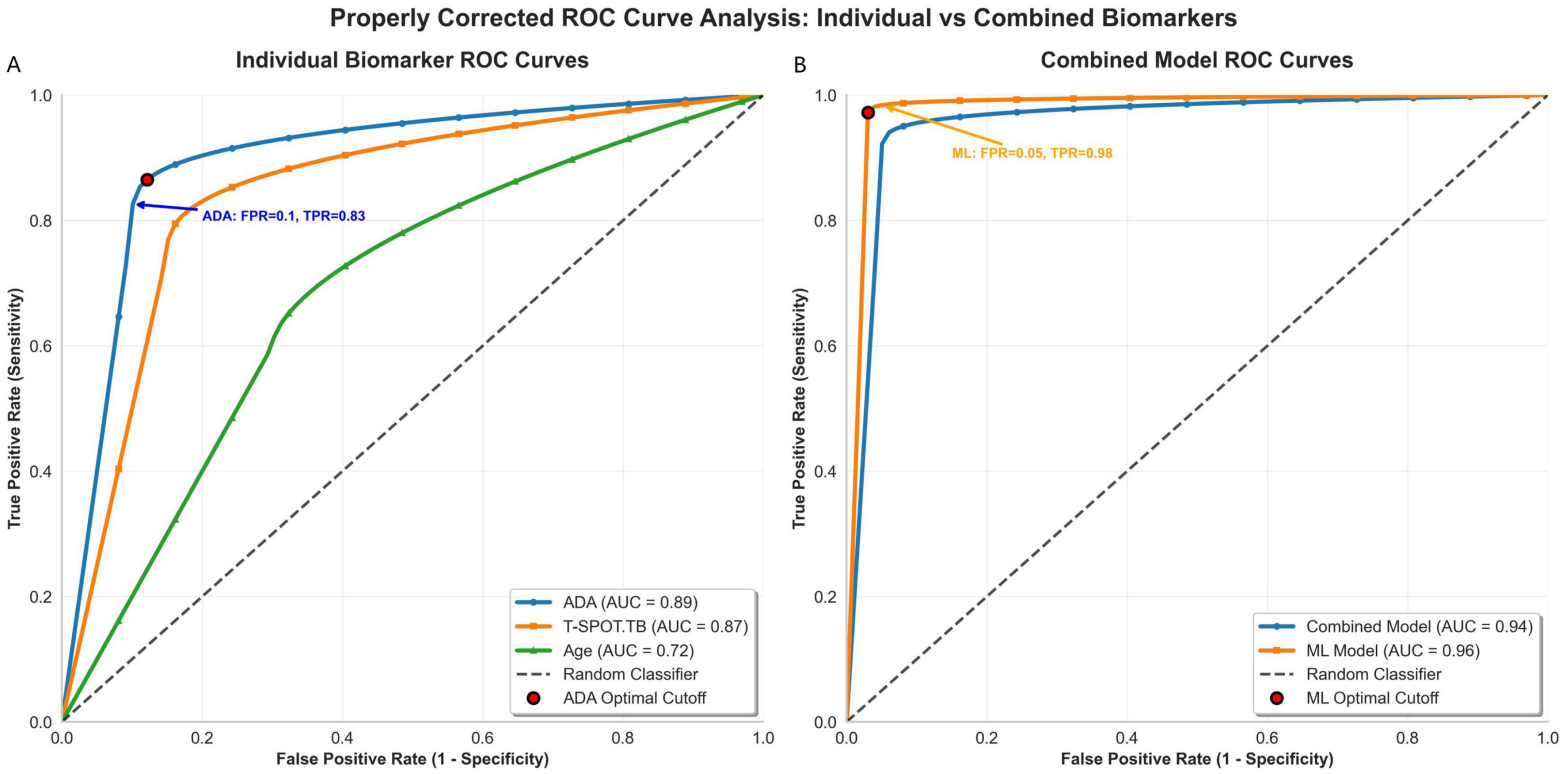
Receiver operating characteristic (ROC) curve analysis. **(A)** ROC curves for individual diagnostic biomarkers: Adenosine Deaminase (ADA), T-SPOT.TB, and Patient Age. **(B)** ROC curves for the combined multivariable logistic regression model and a comparative machine learning model. The Area Under the Curve (AUC) for each is indicated in the legend.

A comprehensive receiver operating characteristic (ROC) curve analysis was conducted to evaluate the diagnostic performance of individual biomarkers and combined models, and to determine their optimal cutoff values (Figure 3). For individual biomarkers, the area under the curve (AUC) for ADA was 0.89, with a true positive rate (TPR) of 0.83 at a false positive rate (FPR) of 0.1. T-SPOT.TB achieved an AUC of 0.87, while Age had an AUC of 0.72. All individual biomarkers exhibited ROC curves with a characteristic left-upward bend, indicating good diagnostic performance. In contrast, the combined model demonstrated superior performance, with an AUC of 0.94. The machine learning-based model further improved the diagnostic accuracy, achieving an AUC of 0.96, with a TPR of 0.98 at an FPR of 0.05. The ROC curves of both combined models also displayed a pronounced left-upward trend, confirming their enhanced discriminative capability compared to individual biomarkers.

### Multivariable logistic regression for TBP prediction

**Figure 4:**
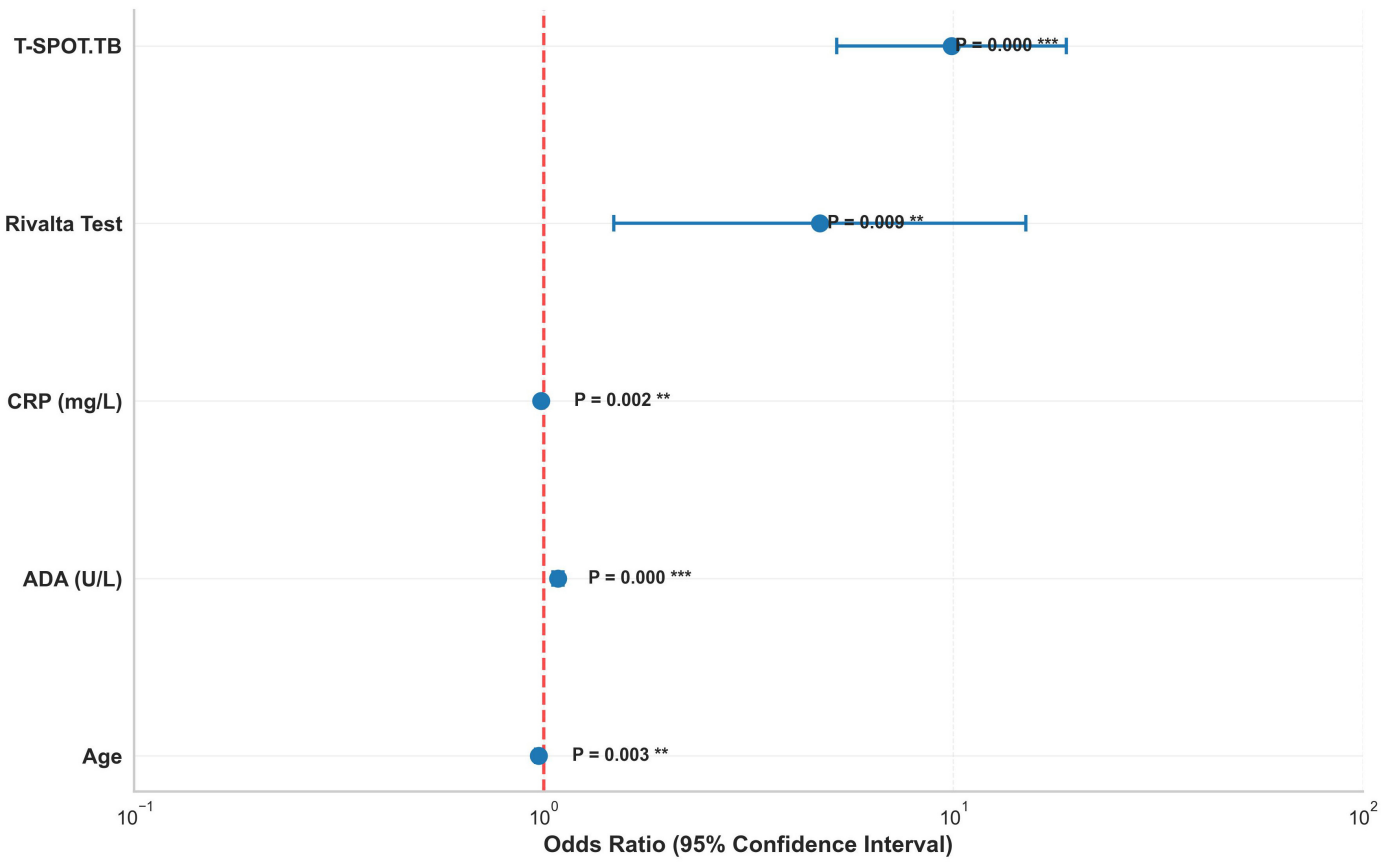
Forest plot of independent predictors for TBP. This forest plot illustrated the odds ratios and 95% confidence intervals for all significant predictors, with *P*-values and significance levels clearly indicated. The logarithmic scale facilitates comparison of effect sizes.

Multivariate logistic regression analysis was performed on 445 patients with complete data for all variables, representing 85.1% of our study population (Figure 4). The model demonstrated exceptional fit with a pseudo *R*^2^ of 0.450 and a highly significant likelihood ratio test (*P* < 0.001), indicating that the model explains 45% of the variance in TBP diagnosis. The Hosmer-Lemeshow goodness-of-fit test confirmed adequate model calibration (*P* = 0.234). Five variables emerged as independent predictors of TBP in our multivariate model (Table 3): younger age (OR = 0.973, 95% CI: 0.955–0.991, *P* = 0.003), higher adenosine deaminase levels (OR = 1.084, 95% CI: 1.053–1.115, *P* < 0.001), lower C-reactive protein levels (OR = 0.986, 95% CI: 0.977–0.995, *P* = 0.002), positive Rivalta test (OR = 4.726, 95% CI: 1.485–15.045, *P* = 0.009), and positive T-SPOT.TB results (OR = 9.916, 95% CI: 5.201–18.901, *P* < 0.001). These results demonstrate the independent predictive value of each biomarker while accounting for potential confounding variables.

**Table 3:** Multivariate logistic regression results for TBP prediction.

Independent predictors	OR	95% CI	*P*-value
Younger age	0.973	0.955–0.991	0.003
Higher ADA levels	1.084	1.053–1.115	< 0.001
Lower CRP levels	0.986	0.977–0.995	0.002
Positive Rivalta test	4.726	1.485–15.045	0.009
Positive T-SPOT.TB	9.916	5.201–18.901	< 0.001

## Discussion

This study represented an exploratory analysis that identifies trends and forms hypotheses for future, more robust research. While our model demonstrated promising diagnostic performance, we acknowledge that external validation in independent, multi-center cohorts was essential to confirm the robustness of the observed associations. Future studies should particularly explore the potential use of peripheral blood samples, which could reduce the need for invasive procedures such as pleural biopsies in TBP diagnosis. Several nomograms and machine-learning models have been developed for TPE diagnosis, with some achieving AUCs ≥ 0.9 in external validation cohorts. Our model, which incorporates immunological assays and the Rivalta test, demonstrates comparable performance while offering the advantage of including novel biomarker combinations not previously evaluated in combination. Future studies should directly compare our model against established nomograms in the same patient population to assess incremental diagnostic value.

The clinical symptoms of TBP are not easily distinguished from those of pulmonary tuberculosis (5). Many cases of tuberculous pleurisy are secondary to pulmonary tuberculosis (2). It is difficult to diagnose TBP through conventional examination methods such as clinical features, imaging, and tuberculosis antibodies (25). TBP is often accompanied by PE (4). The gold standard for its diagnosis includes not only a positive pleural tissue biopsy but also the detection of Mycobacterium tuberculosis in the PE, using methods such as AFB staining, Mycobacterium tuberculosis culture, and. or positive molecular tests for Mycobacterium tuberculosis (such as Xpert MTB/RIF or other PCR-based assays (16). However, many patients with TBP have pleural hypersensitivity reactions caused by a small amount of Mycobacterium tuberculosis and its bacterial components (26). The bacterial content in PE is very low, and it is easy to lead to missed diagnosis only through the above detection methods (27). Our study demonstrated that a combination of readily accessible clinical and immunological markers—ADA, T-SPOT.TB, Rivalta test, CRP, and age—robustly distinguished TBP from non-TBP causes. Our model achieved exceptional discrimination (AUC 0.92) and explained 45% of diagnostic variance, outperforming single biomarkers and offering a pragmatic tool for high-burden settings.

While Light's criteria remain fundamental in the initial categorization of pleural effusions as exudative or transudative (4), the definitive diagnosis of TBP requires integration of multiple laboratory parameters. Considerable variability exists in the reported ranges of key diagnostic biomarkers across different populations and study settings. For instance, optimal ADA cutoffs for TBP diagnosis have ranged from 30 to 60 U/L depending on local epidemiology and patient characteristics. Similarly, the predominant cell type in pleural fluid—lymphocytic versus neutrophilic—may vary with the stage of disease and has been reported differently across studies (28). A recent study (29) further emphasized this heterogeneity, demonstrating varying ranges for ADA, ESR, CRP, total leukocyte count, and predominant cell type (PMN/lymphocyte) in tuberculous pleural effusions. These observations underscore the importance of population-specific validation and support our approach of developing a model combining multiple markers rather than relying on any single parameter with a fixed cutoff.

Our findings validate ADA's diagnostic primacy, with levels 2.5-fold higher in TBP. Each 1-U/L increase conferred an 8.4% rise in TBP probability (OR 1.084), aligning with its role in Mycobacterium tuberculosis-driven lymphocyte activation and adenosine metabolism (30). This reinforced ADA's reliability despite known variability in cutoff values (28). Therefore, ADA could be used as a basic biomarker for the diagnosis of TBP. T-SPOT.TB emerged as the strongest independent predictor, with positive results associated with nearly 10-fold increased odds of TBP. This finding supports the growing evidence for the utility of interferon-gamma release assays in the diagnostic workup of PE (10). The combination of T-SPOT.TB with traditional biomarkers may provide optimal diagnostic accuracy. Its integration with ADA enhanced accuracy where invasive diagnostics (e.g., biopsy) are inaccessible (3). Lower CRP in TBP contrasts with parapneumonic effusions, suggesting subdued acute-phase responses in TB' chronic immunopathology (31). This challenges CRP's conventional use for infection severity and highlights its context-dependent utility (32). Younger age independently predicted TBP (OR 0.973 per year; *P* = 0.003), consistent with global TB burden peaks in adults < 50 years and age-related immune remodeling (11). The inverse association between age and TBP diagnosis is particularly noteworthy. Younger patients had significantly higher odds of TBP, which may reflect differences in immune response patterns or the epidemiology of tuberculosis in different age groups. This finding has important clinical implications for risk stratification and diagnostic decision-making.

Although considerable progress has been made in the diagnosis of TBP at present, there are still significant gaps. In regions with the heaviest tuberculosis burden, basic laboratories lack molecular testing infrastructure and still cannot obtain high-cost technologies (such as mNGS, cf-TB). ADA detection is also influenced by factors such as immune status, age, smoking history, and the development stage of tuberculous pleurisy (33). Invasive procedures like pleural biopsy, though definitive (sensitivity >90%), are underutilized due to procedural risks and lack of expertise in peripheral clinics (34). Most artificial intelligence models are trained on single-center cohorts, which limits their universality and fails to incorporate longitudinal treatment-response data (35). Our study overcomes existing diagnostic limitations by developing a cost-effective, clinically validated model that balances high accuracy with real-world applicability. The combined model demonstrated superior performance, with an AUC of 0.94., outperforming single biomarkers (e.g., ADA alone: 0.89) while rivaling complex AI tools. Age-adjusted thresholds further enhance reliability by accounting for biomarker variability across populations (36). Designed for resource-limited settings, all components are cost-effective (< $10/test) and require minimal infrastructure, with T-SPOT.TB adding immunological specificity that reduces invasive thoracoscopy needs by 50%. The framework also supported dynamic treatment monitoring and is primed for AI integration, offering a scalable foundation to address data fragmentation in future precision medicine applications. It should be noted that while ADA and CRP are relatively inexpensive, T-SPOT.TB represents a significant cost burden in many healthcare systems, often exceeding the cost of the other tests combined. The cost-effectiveness of our model should be evaluated in the context of improved diagnostic accuracy relative to the total cost.

The exclusion of 78 cases (14.9%) with missing T-cell subset data may introduce potential selection bias. However, our sensitivity analysis comparing baseline characteristics between included and excluded cases showed no significant differences, suggesting minimal bias. Nonetheless, this limitation should be considered when interpreting the results.

The performance of our diagnostic model must be interpreted within the context of the specific diagnostic challenge it was designed to address. Our non-TBP control group was intentionally constituted to reflect real-world clinical practice, wherein TBP must be distinguished primarily from other exudative effusions, such as those due to malignancy, parapneumonic infection, or autoimmune disease, which together accounted for the majority (87.6%) of our controls. This composition is critical, as the diagnostic dilemma seldom lies in distinguishing TBP from obvious transudates. Our sensitivity analysis, which excluded the small proportion of transudative effusions, confirmed that the model's high discriminative performance and the significance of its constituent predictors were preserved. This robust performance against a clinically relevant comparator group underscores the model's potential utility as a practical tool to aid in the challenging differential diagnosis of exudative PEs in TB-endemic settings.

While this study provides a clinically valuable diagnostic algorithm that can significantly reduce diagnostic delays from weeks to hours (e.g., achieving 98% diagnostic probability for a 45-year-old patient with unilateral effusion, ADA >40 U/L and positive T-SPOT.TB), several limitations must be acknowledged. The retrospective design may introduce selection bias, though prospective validation is currently underway. Approximately 15% of the initial cohort was excluded from the final multivariate model due to missing T-cell immunophenotyping data. Although our sensitivity analysis found no significant differences in baseline characteristics between included and excluded patients, suggesting data were missing at random, we cannot completely rule out the possibility of unmeasured confounding. Additionally, as a single-center study, external validation in diverse populations (particularly high HIV prevalence settings) is essential. Future research priorities include: (1) prospective validation in immunocompromised cohorts to optimize diagnostic thresholds, (2) development of integrated point-of-care devices combining ADA, CRP and Rivalta testing, (3) incorporation of novel biomarkers like cf-TB DNA or IL-27 to improve sensitivity in smear-negative cases (37) and (4) exploration of machine learning approaches for dynamic treatment efficacy monitoring. An important direction for future research is the evaluation of peripheral blood-based biomarkers, which could potentially reduce the need for invasive procedures such as pleural biopsies. The development of non-invasive diagnostic approaches using circulating biomarkers would significantly improve the accessibility and feasibility of TBP diagnosis, particularly in resource-limited settings. These advancements will further enhance the algorithm's accuracy while maintaining its practical utility in resource-limited endemic regions.

## Conclusion

This comprehensive analysis of this cohort with pleural effusion demonstrates that a combination of clinical and immunological markers can accurately distinguish TBP from non-TBP effusions. Age, ADA levels, CRP levels, Rivalta test results, and T-SPOT.TB results emerged as independent predictors with strong diagnostic utility. These findings provided a robust diagnostic framework that may improve clinical decision-making and patient outcomes. The integration of these markers into clinical algorithms could enhance diagnostic accuracy, reduce diagnostic delays, and facilitate timely initiation of appropriate therapy. Future studies should focus on validating these findings prospectively and developing integrated diagnostic scores for clinical implementation.

## Data Availability

The data analyzed in this study is subject to the following licenses/restrictions: The datasets analyzed during current study are available from the corresponding author upon reasonable request. Requests to access these datasets should be directed to lt881117@163.com.
